# Clinical evaluation of recombinant antigen ELISAs and broad-range helminth PCRs for the diagnosis of human paragonimiasis in Gabon

**DOI:** 10.1186/s41182-026-01083-6

**Published:** 2026-09-24

**Authors:** Stefan Fabian Weber, Rima Jeske, Bayodé Roméo Adegbite, Anne Glaser, Paul A. Nguema Moure, Jenny Mouloungui-Mavoungou, Muriel Rabone, Aidan M. Emery, N’da Angbélétchi David Aka, Heike Jung, Cindy Büchler, Emilija Sorgho-Mitreska, Frank Tobian, Laura Plutowski-Wrobel, Markus Ganter, Michael Lanzer, Thirumalaisamy P. Velavan, Martin P. Grobusch, Claudia M. Denkinger, Tim Waterboer, Ayola Akim Adegnika, Sabine Bélard

**Affiliations:** 1 Medical Faculty, Centre for Infectious Diseases, Department of Infectious Disease and Tropical Medicine, Heidelberg University, University Hospital Heidelberg, Im Neuenheimer Feld 324, 69120 Heidelberg, Germany; 2 Medical Faculty, Centre for Infectious Diseases, Department of Parasitology, Heidelberg University, Heidelberg, Germany; 3 German Center for Infectious Disease Research (DZIF), Partner Site Heidelberg, Heidelberg, Germany; 4 Division of Infections and Cancer Epidemiology, German Cancer Research Center (DKFZ), Heidelberg, Germany; 5 Centre de Recherches Médicales de Lambaréné (CERMEL), Lambaréné, Gabon; 6 Institute of Tropical Medicine, Tübingen University Hospital, Tübingen, Germany; 7 German Center for Infectious Disease Research (DZIF), Partner Site Tübingen, Tübingen, Germany; 8 Center of Tropical Medicine and Travel Medicine, Amsterdam University Medical Centers, location University of Amsterdam, Amsterdam, The Netherlands; 9 Life Sciences Department, Natural History Museum London, London, UK; 10 Université Félix Houphouët-Boigny, Abidjan, Côte d’Ivoire; 11 Vietnamese German Center for Medical Research, Hanoi, Vietnam; 12 Faculty of Medicine, Duy Tan University, Da Nang, Vietnam

**Keywords:** Paragonimiasis, *Paragonimus* spp., Diagnosis, Recombinant serology, PCR, Gabon

## Abstract

**Background:**

Paragonimiasis is a neglected food-borne trematode infection endemic to parts of Africa, Asia, and the Americas acquired by raw/undercooked freshwater crab consumption. Pulmonary manifestations mimic pulmonary tuberculosis (TB). Diagnosis relies on microscopy, with low sensitivity. Development of molecular assays is constrained by an absence of genomic data for African *Paragonimus* species. Serological testing depends on crude worm antigen, resulting in restricted availability and reproducibility.

**Methods:**

This study was nested within a prospective cohort of adults with presumed TB in Lambaréné, Gabon. The reference standard for paragonimiasis consisted of sputum and stool microscopy. Molecular index tests included PCRs (ITS2, cox1) on sputum and stool, followed by sequencing. Serological index tests consisted of recombinant ELISAs using GST-tagged Myo-1, MDP, and Egg antigens. Diagnostic accuracy was evaluated using positive control sera (three individuals with confirmed *P. westermani* infection). Stool, urine and blood were examined for potentially serologically cross-reactive parasites.

**Results:**

Among 103 participants (enrolled September 2024–January 2025), no *Paragonimus* eggs were detected. PCR assays amplified only non-*Paragonimus* sequences, including other helminths. Control sera (confirmed *P. westermani*) showed marked reactivity, particularly toward Myo-1 and Egg antigen, background reactivity in participants was generally low. Without confirmed cases of African paragonimiasis, we could only calculate analytical sensitivity for *P. westermani*-sera. Exploratory cut-offs resulted in tentative diagnostic sensitivity between 17 and 100%. Exploratory cut-offs yielded specificities of 76–100%, assuming that no infections were missed by the reference standard. No relevant cross-reactivity with other infections was observed.

**Conclusions:**

In this first real-life application of recombinant antigen-based ELISAs for the serodiagnosis of paragonimiasis, our results showed promise, particularly for Myo-1 and Egg antigen. These results require further validation in larger and diverse populations including cases with African *Paragonimus* species to better define the epidemiology and burden of paragonimiasis in Gabon and beyond.

**Supplementary Information:**

The online version contains supplementary material available at https://doi.org/10.1186/s41182-026-01083-6.

## Background

Paragonimiasis is a foodborne parasitic disease caused by trematodes of the genus *Paragonimus*. It remains a public health concern in endemic regions of Asia, the Americas, and Africa. Human infection occurs through the consumption of raw or undercooked freshwater crabs containing *Paragonimus* metacercariae, leading to pulmonary and potentially extrapulmonary manifestations [1]. Pulmonary symptoms, such as cough and hemoptysis, may mimic pulmonary tuberculosis (TB, PTB), often resulting in inadequate empiric TB treatment [2]. Gabon and the immediate area around Lambaréné were found to be endemic for *Paragonimus africanus* and *P. uterobilateralis* in surveys from the twentieth century. The last microbiologically confirmed human case was reported > 3 decades ago [3–5]. Our group recently identified *P. uterobilateralis* metacercariae in freshwater crabs within approximately 60 km of the study area, confirming an active local transmission cycle (Weber and Rabone et al. submitted for publication). The current burden of human paragonimiasis in Gabon has not been investigated in recent decades. However, in neighboring Cameroon, paragonimiasis was identified in approximately 3% of patients investigated for presumed PTB and 10% in an active case finding study [6, 7], supporting the need to ascertain the local burden.

Despite its global distribution, the true burden of paragonimiasis is difficult to ascertain due to diagnostic challenges: Direct diagnosis relies on microscopic detection of *Paragonimus* eggs in sputum or stool, methods requiring parasitological laboratory infrastructure with unclear sensitivity, particularly in light infections or sputum-scarce individuals, which limits applicability for diagnostic or epidemiological investigations. Proof-of-principle molecular assays have previously been applied, using broad-range cox1 and ITS2 PCRs, followed by sequencing in known positive samples [7]. Robust accuracy data on these PCR targets are lacking and the development of targeted PCRs specifically for African paragonimiasis is further complicated by the scarcity of genomic data (for *P. africanus* only three and for *P. uterobilateralis* only a single nucleotide sequence were registered at NCBI, no genome was available, access date: September 17, 2026) [8].

Serological testing using crude worm antigens in ELISA, western blot, or indirect hemagglutination assays has supported diagnosis for decades (e.g., [9–11]), but reliance on parasite material for assay production raises concerns about reproducibility, particularly in endemic countries with limited resources. Moreover, cross-reactivity across diverse *Paragonimus* species (e.g., *P. westermani* in Asia, *P. africanus* and *P. uterobilateralis* in Africa [12]) has not been systematically assessed, and accuracy data including assessment for cross-reactivity with other parasitic infections is limited. Cumulative work in recent years has identified candidate pan-*Paragonimus* antigens by applying comparative transcriptomics for recombinant serological assays that may overcome these barriers of reproducibility and increase access to serological diagnostics [13–15]. Preliminary accuracy data exists for several promising antigens like myoglobin (Myo-1), microbial defense protein (MDP) [16], major egg antigen (Egg antigen) [17], and CE3 [18], which warrant further validation in at-risk populations. None of these assays have been tested in a real-life scenario in an at-risk population for paragonimiasis.

The glutathione S-transferase (GST) tag recombinant antigen platform is widely used for production of proteins for serological assays such as ELISA. Candidate antigens are genetically fused to a GST tag, enabling selective immobilization in glutathione-coated ELISA wells to bind antigen-specific antibodies in patient sera. Recombinant antigen platforms offer advantages over traditional crude antigen assays by eliminating reliance on parasite material and reducing cross-reactivity to off-target epitopes, making it a promising research focus to improve access to diagnostics for neglected diseases like paragonimiasis [19, 20].

As discussed above, paragonimiasis remains a relevant differential diagnosis in co-endemic regions for TB and paragonimiasis [3, 21], but its current regional prevalence in Gabon remains unknown. This study addressed a distinct diagnostic question on paragonimiasis and was nested within a larger study of hospitalized adults with presumed PTB which investigated alternative infectious causes of TB-like illness; therefore, the study was conducted at the Centre de Recherches Médicales de Lambaréné (CERMEL), the regional TB reference laboratory.

The aim of this exploratory sub-study was to assess the diagnostic performance of ELISA assays using recombinant *Paragonimus* antigens and previously published broad-range PCR targets (cox1, ITS2) for paragonimiasis.

## Methods

### Setting and selection

This prospective observational diagnostic accuracy study was conducted at two regional hospitals in Lambaréné, Gabon (Hôpital Albert Schweitzer; Centre Hospitalier Régional Georges Rawiri de Lambaréné). This exploratory analysis was nested within a prospective cohort of adults with presumed PTB.

Hospitalized adult patients were screened consecutively in the respective departments of internal medicine for inclusion criteria by chart review or by conversation with healthcare providers. Inclusion criteria were: (1) presumed PTB based on clinical or radiological findings with routine TB workup initiated by attending physicians; and (2) a positive WHO symptom screen (cough lasting 2 weeks or longer [any duration in people living with HIV (human immunodeficiency virus)], fever, weight loss, night sweats [22]). Patients were excluded if they had already taken anti-TB treatment (ATT) for 7 days or longer.

### Study procedures

Consenting participants underwent the following study procedures: a study questionnaire investigated participants’ potential exposure to paragonimiasis (freshwater crustacean (mainly crab) consumption, manner of preparation) and their residence (urban or rural), socioeconomic status (SES, method and details, see [23]). Participants were asked for a blood, a stool, urine and sputum specimen (one spot sputum, and one additional sputum sample collected over > 12 h [long-term sputum]). Study procedures for the primary study outcome (cohort analyses and tests for non-TB pathogens) is beyond the scope of this analysis and will be reported separately (cf. study registry DRKS00034074 and [23]).

### Reference standard for paragonimiasis detection

Long-term sputum and stool samples were assessed for presence of eggs of *Paragonimus* sp. using sedimentation techniques, adapted from [24]. In short, long-term sputum was homogenized with an equal amount of 3% NaOH and centrifuged; stool was sedimented in 200 ml normal saline. Stool and sputum sediments underwent microscopy at the parasitology laboratory of CERMEL. Additionally, ethanol-preserved long-term sputum sediment and a separate stool aliquot after formol-concentration technique underwent repeat microscopy at University Hospital Heidelberg (UKHD). Protozoa were assessed under 100× magnification without staining. DNA was extracted from stool (QIAamp Fast DNA Stool Mini Kit) and sputum (QIAamp DNA Blood Mini Kit) using lambda phage DNA as an extraction control (Lambda Phage DNA, TIB Molbiol); primer forward: ATGCCACGTAAGCGAAACA; probe: ACCTTACAATCGGTACGGATACCGC; primer reverse: CATAAACGAAGCAGTCGAGT).

To detect *Paragonimus* DNA in stool and spot sputum DNA, we used broad-range trematode primers targeting ITS2 and cox1 regions [7], using the following conditions: 50 µl reactions contained forward and reverse primers at 10 µM each in LightCycler 480 Probes Master (Roche) and 4 µl target DNA; cycling conditions for ITS2 were: incubation (95 °C, 7 min), and 40 cycles (95 °C, 10 s; 62 °C, 45 s; 72 °C, 15 s); for cox1: incubation (95 °C, 7 min), and 40 cycles (95 °C, 30 s; 58 °C, 45 s; 72 °C, 30 s). DNA extracted from *P. uterobilateralis* metacercariae and *P. westermani* adults served as positive controls. PCR products were analyzed by standard gel electrophoresis; positive amplicons were purified and underwent Sanger sequencing (Eurofins genomics) after purification with the NucleoSpin® Gel and PCR Clean-up kit (Macherey–Nagel). Sequences were matched to International Nucleotide Sequence Database Collaboration entries via NCBI Nucleotide BLAST searches [25]. All *Schistosoma* spp.-positive samples additionally underwent *S. haematobium* and *S. mansoni*-targeted PCR for species confirmation at UKHD.

### Potential cross-reactive infections

To assess for potential cross-reactive infections, in addition to incidental detection of non-*Paragonimus* parasites by stool/sputum microscopy and ITS2/cox1 sequencing, a thin blood smear was examined for filariae. A spot urine sediment obtained after centrifugation was microscopically examined for presence of *Schistosoma haematobium* eggs and underwent dra1 (*S. haematobium*) PCR at UKHD [26, 27]. *Plasmodium* sp., while being a protozoan, was also assessed for by microscopy for potential effects on specificity associated with polyclonal B-cell stimulation [28]. 

### Index test: recombinant serology

We selected four antigens based on their previously published capacity to detect genus-specific *Paragonimus* antibodies: CE3 (GenBank KX180136 [18]), Myo-1 and MDP (GenBank PK120808, MK050850 [16]), and (Major) Egg-antigen (GenBank Q6QT19 [17]). Protein sequences were obtained from UniProt, and OligoAnalyzer^™^ (Integrated DNA technologies) was used for backtranslation and codon optimization. DNA sequences were subcloned (Eurofins genomics) into a pGEX-4T3tag vector plasmid encoding an N-terminal GST, as well as a C-terminal tag epitope (*simian virus 40* large T-antigen) [29]. Expression was performed at the German Cancer Research Center (Deutsches Krebsforschungszentrum, DKFZ) Heidelberg using transformed *Escherichia coli* BL21. DNA sequence and full-length translation were verified for all proteins by plasmid sequencing, western blot and anti-tag ELISA characterization. Diagnostic monoplex ELISAs were performed after coating wells with each antigen by glutathione linkage. Reactivity was quantified using goat anti-human-IgG/IgM/IgA-pox conjugated reaction, optical density (OD) extinction was measured at 450 nm.

Preclinical analyses involved testing of sera of parasitologically confirmed infections with *S. mansoni* (*n* = 3), *Loa loa* (*n* = 3), *Strongyloides stercoralis* (*n* = 2), *Taenia solium* (neurocysticercosis; *n* = 1), serologically diagnosed cases of *Fasciola* spp. (*n* = 2), *Trichinella spiralis* (*n* = 1), *Toxocara* sp. (*n* = 1) and sera from individuals without exposure to helminths (*n* = 7). Serum from three egg-positive *P. westermani* cases served as reference (positive controls). Antigens were initially tested with individual reference sera; subsequently, positive controls were pooled (1:1:1) to economize available serum.

For the clinical study on stored sera from Gabonese study participants, only Myo-1, MDP, and Egg antigen were included; CE3 was discontinued after poor observed signal-to-noise ratio, resulting in poor diagnostic performance in preclinical experiments (see results). Reference sera included the positive control (see above), as well as one negative serum (SW). The assays were first performed at the study site (CERMEL, Gabon) in triplicates at serum dilutions of 1:100, and positive controls at dilutions of 1:100, 1:555, and 1:3080. A confirmatory assay run was conducted at DKFZ Heidelberg using positive controls at dilutions of 1:555, 1:3080 (due to scarcity of sera).

### Data analysis

Missing data are indicated by denominators indicating available data throughout. Measured OD values were analyzed to assess antibody reactivity after subtracting background reactivity to GST, negative net values were set to 0. Initial comparisons were performed visually using boxplots for reference and participant sera. Under the assumption that most participants were negative, cutoffs were defined by calculating the mean + 3/5/10 standard deviations (SD) and removing outliers. This cycle continued until the calculation converged (no more outliers). Sensitivity and specificity with 95% confidence intervals (95%CI) were calculated. For sensitivity, all measurements of the positive sample pool were used, acknowledging limited generalizability. For specificity, participants with negative testing for paragonimiasis were assumed to be negative. Subgroup analyses were conducted for individuals with possible cross-reactive infections and selected patient characteristics, p-values were calculated using Mann–Whitney U test. All analyses and figures were done using R version 4.5.1 (packages: ggplot2, tidyr, dplyr, purrr).

### Ethical considerations

The study was registered under German trial registry number (DRKS00034074) and approved by Institutional Ethics Committees (CERMEL CEI-006/2024; Heidelberg S234/2024). The study was conducted in accordance with Good Clinical Practices and the Helsinki declaration. All participants provided written informed consent.

## Results

### Participant characteristics

From September 2024 to January 2025, 103 participants were recruited and the median age was 44 years (interquartile range, IQR 30.5; 58.5), 61/103 (59%) were female, 30/102 (29%) were HIV-infected (Table 1).

**Table 1:** Participant characteristics

	ALL (*n* = 103)	Urban residence (*n* = 72)	Rural residence (*n* = 31)
Age in years, median (IQR)	44 [30.5;58.5]	38.5 [26;56]	54 [39.5;67]
Sex female, *n* (%)	61 (59)	43 (60)	18 (58)
Urban residence, *n* (%)	72 (70)	72/72 (100)	0/31 (0%)
Eats freshwater crabs, *n* (%)	46 (45)	25 (35)	21 (68)
Eats unsafe preparations of freshwater crabs (e.g., raw, dried, pickled, smoked), *n* (%)	7/46 (15)	3/25 (12)	4/21 (19)
Cough present, *n* (%)	97 (94)	68 (94)	29 (94)
Hemoptysis present, *n* (%)	31 (30)	22 (31)	9 (29)
Laboratory testing and cross-reactive infections			
Eosinophiles (/nl)	0.16 [0.05;0.56] (*N* = 90)	0.16 [0.06;0.57] (*N* = 63)	0.22 [0.05;0.48] (*N* = 27)
Eosinophilia > 0.5/nl, *n* (%)	24/90 (27)	17/63 (27)	7/27 (26)
Paragonimiasis by stool/sputum microscopy or PCR*	0/101 (0)	0/70 (0)	0 (0)
Any parasitic infection*	40/101 (40)	30/70 (43)	10 (32)
Any helminth infection, *n* (%)**	26/100 (26)	20/69 (29)	6 (19)
Any intestinal helminth infection, *n* (%)	19/78 (24)	14/58 (24)	5/20 (25)
*Strongyloides stercoralis*	9/78 (12)	7/58 (12)	2/20 (10)
*S. haematobium*	4/78 (5)	4/58 (7)	0/20 (0)
*Trichuris trichiura*	4/78 (5)	2/58 (3)	2/20 (10)
Hookworm	3/78 (4)	1/58 (2)	2/20 (10)
*S. mansoni*	1/78 (1)	1/58 (2)	0/20 (0)
*Ascaris lumbricoides*	1/78 (1)	0/58 (0)	1/20 (5)
Any schistosomiasis***	13/100 (13)	12/69 (17)	1 (3)
Urinary schistosomiasis***	11/100 (11)	10/69 (14)	1 (3)
Intestinal schistosomiasis***	5/78 (6)	5/58 (9)	0/20 (0)
*Loa loa*	1/101 (1)	1/70 (1)	0 (0)
Intestinal protozoa****	12/77 (16)	7/58 (12)	5/19 (26)
*Plasmodium falciparum (thin blood smear)*	6/101 (6)	6/70 (9)	0 (0)

Denominators provided if differing from figure in column-head; *IQR* interquartile range

^*^denominator: all participants with at least one spot or long-term sputum sample available

^**^denominator: all participants with at least one spot or long-term sputum sample available; additionally at least one stool or urine sample

^***^urinary detection: *S. haematobium* *n* = 11; intestinal detection: *S. haematobium* *n* = 2, *S. mansoni* *n* = 1, *S. haematobium/S. bovis* *n* = 2 (dra1 positive, *S. bovis* by ITS2-sequence alignment)

^***^denominator: at least one urine or stool sample available

^****^The assessment of intestinal protozoa was based on routine light microscopy without dedicated staining or molecular methods and may underestimate the prevalence. Species: *Entamoeba coli* 5/77 (6%), *Endolimax nana* 4/77 (5%), *Chilomastix mesnili* 2/77 (3%), *Giardia lamblia* 2/77 (3%), *Blastocystis hominis* 1/77 (1%), *Cystoisospora belli* 1/77 (1%)

Participants’ residence was urban in 72/103 (70%), and rural in 31/103 (30%). Participants’ main symptom was cough (97/103, 94%) and 31/103 (30%) reported hemoptysis. Most participants (56/100, 56%) reported eating fresh-water crustaceans and 46/100 (46% or 46/56, 82%) reported eating crabs (other crustaceans: crayfish 27/100, 27%, and shrimp 47/100, 47%). Crabs were eaten rarely (< 5 times per year) by 32/46 (70%), occasionally (5–15 times per year) by 7/46 (15%), regularly (16–52 times per year) by 6/46 (13%), and frequently (> 52 times per year) by 1/46 (2%). The most common preparation was boiling (38/46, 83%), followed by frying (15/46, 33%), drying (6/46, 13%), smoking (2/46, 4%), pickling (2/46, 4%), or raw consumption (1/46, 2%), amounting to a total of 7/46 (15%) of unsafe preparations (dried, smoked, pickled or raw).

Complete blood counts were available for 90 participants and showed eosinophilia (> 0.5/nl) in 24/90 (27%).

Serum samples were available for 101, spot sputa for 100, long-term sputa for 96, stool for 78, and urine for 100 participants.

Stool microscopy for helminth infections was negative in 59/78 (76%) and revealed *Strongyloides stercoralis* in 9/78 (12%), *Schistosoma* spp. in 5/78 (6%), *Trichuris trichiura* in 4/78 (5%), hookworm in 3/78 (4%), and *Ascaris lumbricoides* in 1/78 (1%) samples. Stool microscopy for protozoa was negative in 65/78 (83%) and revealed *Entamoeba coli* in 5/78 (6%), *Endolimax nana* in 4/78 (5%), *Chilomastix mesnili* in 2/78 (3%), *Giardia lamblia* in 2/78 (3%), *Blastocystis hominis* in 1/78 (1%) *Cystoisospora belli* in 1/78 (1%) samples.

Urine microscopy or dra1-PCR was positive for *S. haematobium* in 11/100 (11%) cases. Combining intestinal or urinary investigations amounted to 13/100 (13%) participants with schistosomiasis (denominator: at least stool or urine available). Blood smears revealed *P. falciparum* in 6/101 (6%) and *Loa loa* in 1/101 (1%) cases.

In summary, any parasitic infection was seen in 40/101 (40%), see Table 1.

### *Paragonimus* reference standard and *Paragonimus* PCR results

No *Paragonimus* spp. eggs were detected by microscopy in long-term sputum or stool samples.

Extraction controls were positive for all samples. ITS2 PCR was positive by gel electrophoresis in 17/100 (17%) sputum and 23/78 (29%) stool samples. Cox1 PCR was positive in 0/100 (0%) sputum and 2/78 stool (3%) samples. All amplicons were sequenced and no sequence aligned with *Paragonimus* spp., but some incidental findings were observed: in 2/23 (9%) ITS2-positive stool samples, sequences aligned closely with *Schistosoma bovis* (100% identity, e-value: 0.0; in both cases, *Schistosoma* spp. eggs were detected in stool, each with positive dra1-PCR for *S. haematobium*). Of 17 ITS2-positive sputum samples, sequences aligned with *Loa loa* (100% identity, *e*-value: 2 × 10⁻^102^; participant with hemoptysis, positive for microfilariae in blood smear), or *S. mansoni* (100% identity, *e*-value: 1 × 10⁻^125^; confirmed by targeted PCR, no stool sample available) in 1/17 (6%) each. For all other sequences closest alignment was found for a wide variety of eukaryotes (e.g., *Holostephanus* sp., *Mansonia* spp., *Homo sapiens*, *Lepidocyrtus* spp., *Austroglycyphagus* spp.), fungi and bacteria (e.g., *Klebsiella* spp.), unlikely to be related to infection.

### Serology—preclinical validation

Initial testing with three reference sera of confirmed cases with *P. westermani* infection (individually and pooled) showed strong reactivity across antigens at all tested concentrations (Additional file 1, Supplement Fig. 1). Reactivity for negative controls and other tested helminth-infection sera was minimal for Myo-1, and egg-antigen; moderate reactivity was seen for MDP, while maintaining a good signal-to-noise ratio between paragonimiasis and other sera. For CE3, reactivity was seen across all samples including negative controls.

### Serology—clinical cohort

**Fig. 1:**
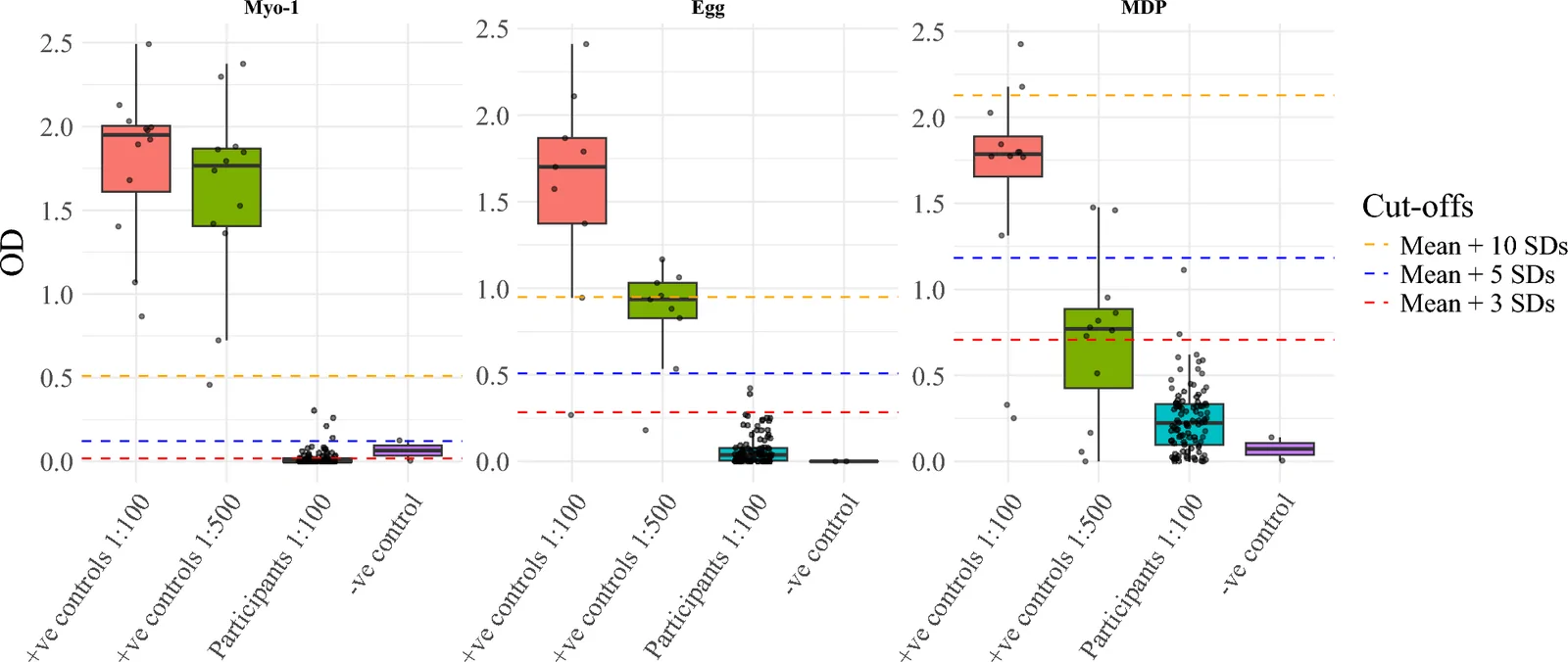
Paragonimiasis ELISA, Gabonese cohort and exploration of potential cutoff values. *SD* standard deviation. Positive controls: sera from patients with microscopy-positive infection with *Paragonimus westermani*. Cutoffs were derived iteratively under the assumption of an absence of paragonimiasis. Low-level true seropositive individuals may have been treated as statistical outliers. Selected thresholds are therefore exploratory and require independent validation

The recombinant antigen ELISA results for the clinical cohort are provided in Table 2 and Fig. 1.

**Table 2:** Serology results and subgroup analyses by demographic variables and infection status

	OD median (IQR)				*p*-value		
	*n*	Myo-1	Egg antigen	MDP	Myo-1	Egg antigen	MDP
Participants*	100	0.00 [0.00;0.02]	0.04 [0.00;0.08]	0.22 [0.10;0.33]	< 0.001	< 0.001	< 0.001
Positive controls 1:100**	12	1.95 [1.61;2.00]	1.70 [1.37;1.87]	1.79 [1.66;1.89]			
Male	39	0.01 [0.00;0.03]	0.04 [0.01;0.10]	0.23 [0.12;0.34]	0.0172	0.0761	0.6208
Female	61	0.00 [0.00;0.01]	0.03 [0.00;0.07]	0.22 [0.06;0.32]			
Age < 44 years (median)	48	0.00 [0.00;0.02]	0.04 [0.00;0.08]	0.22 [0.08;0.32]	0.8612	0.8789	0.5189
Age ≥ 44 years (median)	52	0.00 [0.00;0.02]	0.04 [0.01;0.08]	0.23 [0.14;0.33]			
HIV-infected	30	0.00 [0.00;0.01]	0.04 [0.00;0.08]	0.28 [0.19;0.32]	0.3765	0.5209	0.1652
HIV-uninfected	70	0.00 [0.00;0.03]	0.03 [0.00;0.08]	0.20 [0.08;0.34]			
SES high	25	0.00 [0.00;0.01]	0.03 [0.00;0.13]	0.25 [0.03;0.34]	ND		
SES medium	24	0.00 [0.00;0.01]	0.04 [0.00;0.08]	0.20 [0.14;0.32]			
SES low	29	0.01 [0.00;0.03]	0.04 [0.00;0.08]	0.18 [0.11;0.31]			
Urban residence	70	0.00 [0.00;0.02]	0.04 [0.00;0.08]	0.23 [0.12;0.33]	0.5189	0.6342	0.5153
Rural residence	30	0.00 [0.00;0.01]	0.03 [0.00;0.06]	0.21 [0.04;0.34]			
Any helminth infection***	26	0.00 [0.00;0.01]	0.02 [0.00;0.08]	0.21 [0.08;0.32]	0.3689	0.2974	0.5351
No helminth infection***	73	0.00 [0.00;0.02]	0.04 [0.01;0.08]	0.24 [0.12;0.33]			
Schistosomiasis***	13	0.00 [0.00;0.01]	0.02 [0.00;0.08]	0.18 [0.08;0.21]	0.4008	0.6286	0.1304
No schistosomiasis***	86	0.00 [0.00;0.02]	0.04 [0.01;0.08]	0.24 [0.11;0.34]			
Any parasitic infection***	40	0.00 [0.00;0.01]	0.03 [0.00;0.07]	0.22 [0.09;0.34]	0.0996	0.2409	0.8246
No parasitic infection***	60	0.01 [0.00;0.02]	0.04 [0.01;0.08]	0.23 [0.12;0.32]			

^*^denominator: all participants with at least one spot or long-term sputum sample available

^**^pooled samples tested repeatedly

^***^denominator: all participants with at least one spot or long-term sputum sample available; additionally, at least a stool or urine sample

For Myo-1, visually, there was no overlap between positive controls at 1:100 and 1:500 and participant samples were narrowly distributed. Assuming that all participants were negative for paragonimiasis and applying 3, 5, and 10 SD cut-offs, reactivity against P*. westermani* reference sera was 100%, and specificity ranged between 76 and 100%, see Table 3.

**Table 3:** Positive control detection and tentative specificity estimates by applying study cutoffs

Cut-off	Myo-1 antigen			Egg antigen			MDP antigen		
	3SDs	5SDs	10SDs	3SDs	5SDs	10SDs	3SDs	5SDs	10SDs
Sensitivity (95%-CI)*	1 [0.75;1]	1 [0.76;1]	1 [0.76;1]	0.89 [0.57;0.99]	0.89 [0.57;0.99]	0.78 [0.45;0.94]	0.83 [0.55;0.95]	0.83 [0.55;0.95]	0.17 [0.05;0.45]
Specificity (95%-CI)	0.76 [0.67;0.83]	0.96 [0.90;0.98]	1 [0.96;1]	0.98 [0.93;0.99]	1 [0.96;1]	1 [0.96;1]	0.98 [0.93;0.99]	1 [0.96;1]	1 [0.96;1]

^*^since no cases of African *Paragonimus* species were identified in our cohort, the tentative sensitivity estimates are based on control sera from individuals with *P. westermani* infection and should be interpreted with caution

For the egg antigen, the participant distribution was also narrowly distributed and visually distinguished from the positive controls, where only one outlier had a low reactivity in the range of participant sera (but outside the 1.5xIQR (whisker) range). Sensitivity ranged between 78 and 89% and specificity between 98 and 100%.

For MDP, the participant distribution was broad and visually overlapped with positive controls at 1:500; in positive controls at 1:100, two sera overlapped with MDP. Sensitivity ranged between 17 and 83% and specificity between 98 and 100%.

**Fig. 2:**
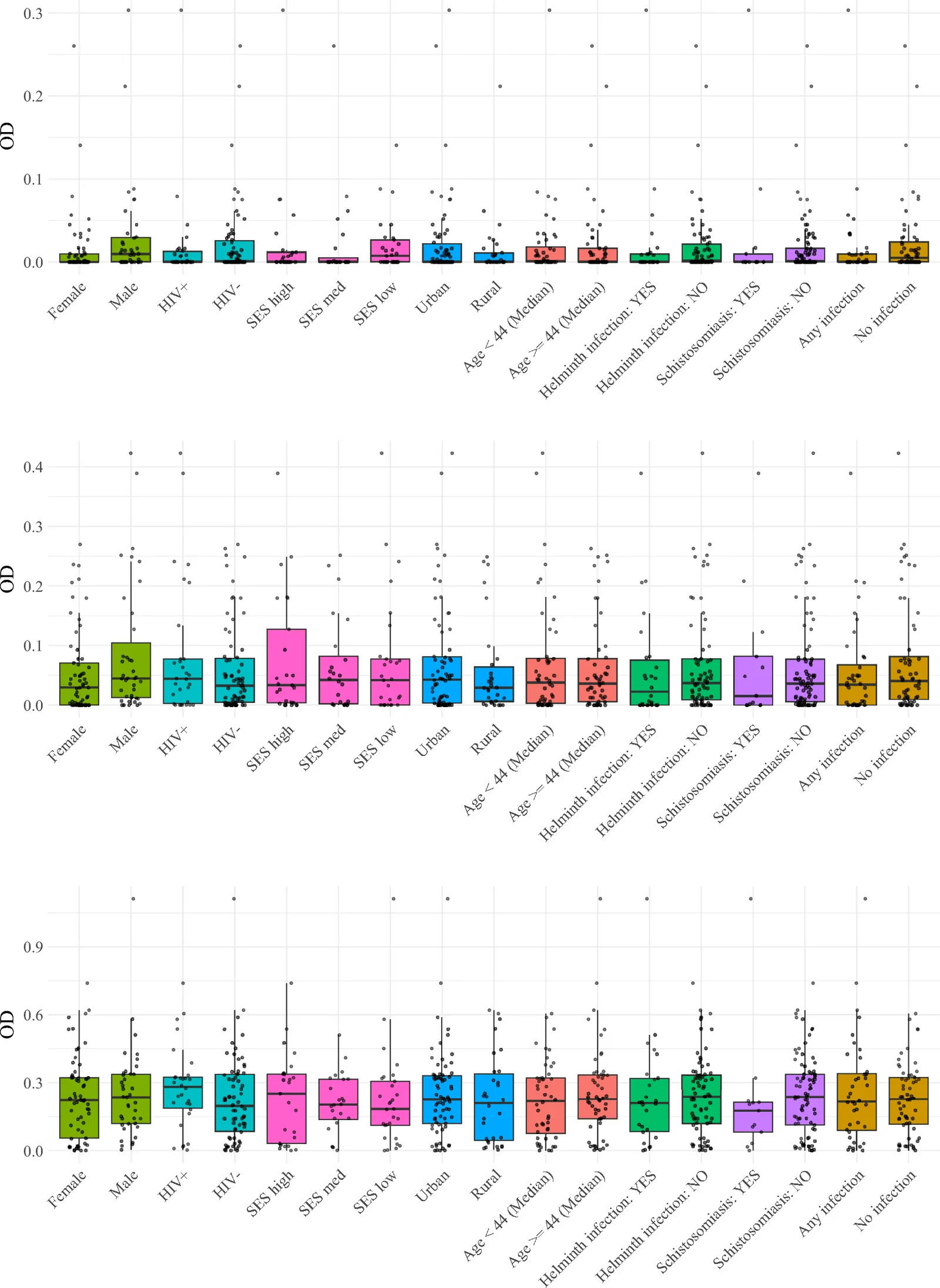
Paragonimiasis ELISA, Gabonese cohort, stratified by demographics and infections. Top: Myo-1, middle: Egg antigen, bottom: MDP. *HIV* human immunodeficiency virus, *SES* socioeconomic status

Stratifying ELISA reactivity by the demographic variables HIV-status, SES, rural/urban residence, and age showed no relevant subgroup differences, see Fig. 2 and Table 2. Although a statistically significant difference between sexes was observed, the absolute difference was minimal and is therefore unlikely to be clinically meaningful. Additional subgroup analyses for potentially cross-reactive infections, similarly, showed no elevated reactivity in participants with or without parasitic infections.

## Discussion

This first prospective and systematic evaluation of recombinant *Paragonimus*-antigen ELISA and broad-range helminth PCR assays for *Paragonimus* spp. detection in sputum and stool from a cohort of adults with presumed PTB in Gabon found promising performance of Myo-1 and Egg antigen for serology, but poor specificity of ITS2 and cox1 PCRs due to off-target amplification.

No case of paragonimiasis could be confirmed in our cohort despite possible exposure being prevalent in the form of 46% of participants eating freshwater crabs, of which 15% reported unsafe preparation methods. The exposure questionnaire was intended to identify potential transmission opportunities rather than to quantify individual infection risk and future studies could incorporate further anthropological methods to better characterize food preparation practices associated with *Paragonimus* transmission. Historically, Gabon and specifically the immediate area surrounding Lambaréné, our study site, has been known to be endemic for *P. africanus* and *P. uterobilateralis* [3, 4, 30], and although reporting on human cases is scarce (the latest microbiologically confirmed case from 1997), an intact transmission cycle of *P. uterobilateralis,* most likely maintained by carnivorous mammals, was confirmed at a more remote location some 60 km away from Lambaréné, indicating the potential for human disease (Weber and Rabone et al. submitted for publication).

Our reference standard consisted of classical microscopy of sputum and stool as well as the application of two investigational sets of broad-range helminth primers. In contrast to a previous study that applied ITS2 and cox1 PCRs only to a small number of selected cases of microscopically confirmed paragonimiasis [7], we tested all available sputum and stool samples. This systematic approach revealed limitations of ITS2/cox1 PCR as a primary diagnostic tool: both primer sets and particularly ITS2 frequently amplified nontarget DNA, including fungi, bacteria, arthropods, and human sequences, indicating limited specificity in clinical material.

Incidental infections detected in stool, blood or urine amounted to 40/101 (40%) of participants and 26/100 (26%) of participants with available stool or urine sample had at least one helminth infection. In previous studies from the region, helminth infections were also common reaching > 30% prevalence [31, 32], which underscores the often polyparasitic burden in the region and the importance of evaluating cross-reactivity in serological assays targeted for use in endemic settings.

Among the evaluated antigens, Myo-1 showed a narrow distribution of signal values in the study cohort and a clear distinction between negative controls and confirmed *P. westermani*–positive reference samples. Applying a conservative positivity threshold (mean + 10SDs), specificity relative to the reference standard was 100%, in line with previous evaluations (100% sensitivity and 100% specificity as per *P. kellicotti* and *P. westermani* reference sera; specificity based on 28 controls, [16]). This separation suggests low background reactivity and highlights Myo-1 as a particularly promising candidate for further validation.

The Egg antigen also showed a narrow distribution of signal values, with only a few visual outliers (all without history of freshwater crab consumption). Specificity was 100%, applying again a mean + 10SD threshold. Previous literature suggested 90% sensitivity and 100% specificity in a small evaluation [17]. These properties also make the Egg antigen a promising candidate for further evaluation.

In contrast, the MDP antigen showed a broader distribution of signal values across the cohort, neither visual nor algorithmic cutoff determination provided good accuracy. While specificity was good (98–100%), the cut-off selection resulted in sensitivities of 16–83% only. This may be due to an unfavorable signal-to-noise ratio and is in contrast with available preclinical data (76% sensitivity and 100% specificity using 17 *P. kellicotti* or *P. westermani* reference sera and 7 control sera [16]) and suggests a limited applicability of MDP as compared to Myo-1 and Egg antigen.

The CE3 antigen, although demonstrating good diagnostic accuracy in preclinical studies (89% sensitivity, 96% specificity [18]), was not included in the clinical evaluation due to observed cross-reactivity with sera from patients infected with other helminths in our preclinical testing. This raises concerns about its specificity in endemic populations with a high burden of co-infections.

Across antigens, elevated reactivity was observed in a small number of samples, and very few outliers reached the range of positive controls. Notably, none of these were consistently positive across all antigens and subgroup analyses by various other helminth infections, HIV-status etc. showed no relevant cross-reactivity. In summary, the observed reactivity may be due to a broad background signal range rather than true exposure to *Paragonimus*.

### Strengths

To our knowledge, this is the first prospective assessment of recombinant *Paragonimus* antigens in a real-world cohort from a region with historical endemicity and evidence of ongoing *Paragonimus* transmission in freshwater crabs. The recombinant serological assays were produced externally, but successfully applied using existing laboratory infrastructure in Gabon, with parallel confirmation in Germany. This demonstrates the practical feasibility of implementing such diagnostic platforms in endemic settings, even without local antigen production, and highlights their potential for use in resource-limited environments.

The diagnostic work-up included both microscopy and PCR for *Paragonimus*, as well as tests for other infections. Although no paragonimiasis cases were identified, over one quarter of participants had at least one helminth infection and 40% had any parasitic infection. This provided an opportunity to evaluate potential background reactivity and cross-reactivity of the recombinant antigens, increasing confidence in the absence of confirmed cases and supporting conclusions on specificity. Once adequate genome sequences for African *Paragonimus* species become available, targeted PCR assays may mitigate the potential concern of missing cases by microscopy alone.

The true burden of paragonimiasis in Gabon cannot be adequately judged based on this proof-of-principle study. Infection risk may be very focal, and low-level prevalence may be missed in this small sample size cohort from the semi-urban Lambaréné region. Moreover, literature suggests that paragonimiasis may disproportionately affect children [33], a population not included in this study. Future studies therefore need to focus on larger cohorts (including children) from various areas in Gabon.

Diagnostic accuracy of ITS2 and cox1 assays for human samples is not well characterized, and especially ITS2 detected many non-*Paragonimus* targets in clinical sputum and stool samples. We therefore cannot exclude that *Paragonimus* DNA was present in some samples but went undetected due to preferential amplification of non-*Paragonimus* sequences. More specific primers, probe-based assays, or nested PCR designs will be required for targeted diagnostic assays, and sequencing data from *P. uterobilateralis* metacercariae may identify optimized targets. Nevertheless, the broad-range PCRs yielded clinically relevant incidental findings, including possible *Schistosoma bovis/S. haematobium* hybrids and *Loa loa* DNA in blood-stained sputum. Such results illustrate that broad-range assays, while unsuitable for screening, may support parasite characterization and reveal unexpected helminths in endemic regions.

### Limitations

Only three sera from confirmed *P. westermani* cases were available as positive controls and served as an analytical reference, demonstrating that recombinant antigens were recognized by patient antibodies. The antigens were selected in view of favorable comparative genomic and cross-species studies suggesting orthologous or conserved candidate diagnostic proteins and likely recognition at genus level [16]. However, these analytical estimates should not be interpreted as reliable estimates for the clinical sensitivity for African paragonimiasis, as cross-species recognition still needs to be demonstrated by future studies.

Although participants originated from a region with historical endemicity and recent evidence of infected intermediate hosts, this cohort was recruited based on presumed PTB rather than residence near a confirmed transmission focus and not designed to estimate the prevalence of paragonimiasis or maximize case detection. Future epidemiological studies should therefore include active case finding in communities surrounding confirmed transmission sites to characterize sensitivity for African species. Future studies need to also investigate the antibody dynamics after past or cured infections longitudinally to account for varying infection intensities and clinical stages. These studies should include differential analyses of immunoglobulin subclasses (IgG, IgA, and IgM) to characterize the ideal use case for these assays, such as tests for sero-epidemiological surveys, individual diagnosis in the absence of confirmatory testing or therapy monitoring.

The pooled infected sera used on each ELISA plate for positive controls showed a moderate distribution range. Ideally, in future iterations or adaptations, a well-defined positive control (e.g., monoclonal antibody, defined concentration) may reduce inter-assay variability.

We did not include a healthy control group, which may strengthen specificity estimates in future studies, but the theoretical additional value is uncertain since all participants tested negative in our *Paragonimus* reference standard, and specificity of most antigens appeared promising despite multiple possible cross-reactive infections.

## Conclusion

Our study highlights the feasibility of a reproducible, recombinant antigen-based serological platform for the neglected tropical disease paragonimiasis. Myo-1 and the Egg antigen, in particular, demonstrated promising performance in a setting co-endemic for various potential cross-reactive helminths, positioning these well for further validation in larger cohorts with confirmed infections.

Our study did not detect human paragonimiasis despite risk-associated eating habits in a region with an intact transmission cycle of *P. uterobilateralis*. ITS2 and cox1 PCR appear suboptimal for diagnostic applications. Larger and spatially expanded surveys are needed to define the true burden of paragonimiasis in Gabon.

## Supplementary Information

Additional file 1.

## Data Availability

The anonymized dataset underlying this analysis will be made available upon reasonable request in accordance with relevant institutional policies governing the use of patient data.

## Abbreviations

ATT: Anti-tuberculosis treatment
BLAST: Basic Local Alignment Search Tool
CEI: Comité d’Éthique Institutionnel (Institutional Ethics Committee)
CERMEL: Centre de Recherches Médicales de Lambaréné
cox1: Cytochrome c oxidase subunit 1 gene
DNA: Deoxyribonucleic acid
DKFZ: Deutsches Krebsforschungszentrum (German Cancer Research Center)
dra1: Schistosoma haematobium-specific DNA repeat sequence (Dra1 PCR target)
ELISA: Enzyme-linked immunosorbent assay
GCP: Good clinical practice
GST: Glutathione S-transferase
HIV: Human immunodeficiency virus
IQR: Interquartile range
ITS2: Internal transcribed spacer 2
MDP: Microbial defense protein
Myo-1: Myoglobin 1 antigen (*Paragonimus* antigen)
NaOH: Sodium hydroxide
NCBI: National Center for Biotechnology Information
OD: Optical density
PCR: Polymerase chain reaction
PTB: Pulmonary tuberculosis
SES: Socioeconomic status
SD: Standard deviation
TB: Tuberculosis
UKHD: Universitätsklinikum Heidelberg (University Hospital Heidelberg)
