# Supplementary material for: Clinical evaluation of recombinant antigen ELISAs and broad-range helminth PCRs for the diagnosis of human paragonimiasis in Gabon
Source: Trop Med Health. 2026 Sep 24;54:209. doi: 10.1186/s41182-026-01083-6 (PMC13602761; doi:10.1186/s41182-026-01083-6)
Supplement: Supplementary file 1 [file 41182_2026_1083_MOESM1_ESM.docx]

**Additional file 1 for**

**Clinical evaluation of recombinant antigen ELISAs and broad-range helminth PCRs for the diagnosis of human paragonimiasis in Gabon**

***Supplement Figure 1*** – paragonimiasis ELISA, pre-clinical validation

A

B

A: reference sera reactivity against CE3, Egg antigen, MDP and Myo-1: individual cases of paragonimiasis, fascioliasis, schistosomiasis, toxocariasis, trichinosis, cysticercosis and uninfected controls; each tested at concentrations of 1:100 and 1:1000

B: reference sera reactivity against Egg antigen, MDP and Myo-1: pooled paragonimiasis serum (1:1:1 of individual sera) at dilutions of 1:1000 and 1:4000; individual samples of strongyloidiasis, *Loa loa* infections and uninfected controls
